# Editorial: Thyroid hormone actions in cancer, volume II

**DOI:** 10.3389/fendo.2025.1652504

**Published:** 2025-07-08

**Authors:** Florencia Cayrol, Helena Andrea Sterle, Maria Del Mar Montesinos

**Affiliations:** ^1^ Laboratorio de Neuroinmunomodulación y Oncología Molecular, Instituto de Investigaciones Biomédicas (BIOMED)-Consejo Nacional de Investigaciones Científicas y Técnicas (CONICET)-Universidad Católica Argentina (UCA), Buenos Aires, Argentina; ^2^ Centro de Investigaciones en Bioquímica Clínica e Inmunología - Consejo Nacional de Investigaciones Científicas y Técnicas (CIBICI-CONICET), Departamento de Bioquímica Clínica, Facultad de Ciencias Químicas, Universidad Nacional de Córdoba, Cordoba, Argentina

**Keywords:** hyperthyroidism, hypothyroidism, cancer risk, cancer progression, epigenetic regulation

Thyroid hormones (THs), primarily triiodothyronine (T3), and thyroxine (T4) are key regulators of cellular metabolism, proliferation, and differentiation. Their canonical actions are mediated by nuclear thyroid hormone receptors (TRα and TRβ), which function as ligand-dependent transcription factors (1). Beyond these classical genomic effects, THs also exert non-genomic actions through integrin αvβ3, located on the cell membrane, triggering intracellular signaling cascades such as MAPK/ERK and PI3K/AKT pathways, which are both known to be involved in cancer progression and resistance to therapy. These non-canonical effects have been increasingly recognized as pro-tumoral in various malignancies (2, 3). Recent studies have demonstrated that THs can promote angiogenesis, cell migration, and proliferation in a range of tumor types, including gliomas, hepatocellular carcinoma, and breast cancer (3, 4). The role of thyroid hormones extends beyond their traditional function as regulators of metabolism to include critical influences on tumor growth and progression. This paradigm shift has opened new avenues of investigation into the endocrine regulation of cancer and has raised crucial questions about the role of systemic thyroid status in oncogenesis (5–7). In addition, alterations in thyroid status have been shown to influence cancer onset, progression, and patient prognosis. Moreover, emerging evidence suggests that tumor cells themselves may actively disrupt both local and systemic homeostasis by releasing neurohormonal and immune signals. These mediators can impact central neuroendocrine organs—including the hypothalamus, pituitary gland, adrenals, and thyroid—potentially creating a bidirectional interaction between the tumor and the endocrine system (8).

Based on these foundational insights, this Research Topic applies Mendelian randomization (MR) to dissect the causal relationships between genetically predicted thyroid dysfunction and the risk of various gastrointestinal and hepatobiliary malignancies. These studies employed independent MR designs, which collectively illustrate a cohesive and compelling picture: thyroid hormone imbalance, whether due to excess, deficiency, or subclinical variation, may influence cancer development through both direct hormonal signaling and indirect metabolic pathways. This integrated evidence highlights the multifaceted role of thyroid hormones in modulating oncogenic processes and underscores the importance of considering thyroid status in cancer risk assessment and potential therapeutic strategies.

Zhu et al. demonstrated through two Mendelian randomization studies that hyperthyroidism has a dual causal effect: it is associated with a decreased risk of gastroesophageal reflux disease (GERD), but at the same time, it shows a slight but significant increase in the risk of esophageal cancer. This divergence highlights the complex and context-dependent role of thyroid hormones in gastrointestinal physiology and tumorigenesis. Notably, there was no evidence of a reverse causal relationship from genetic susceptibility to hyperthyroidism toward GERD or esophageal cancer. These findings provide robust genetic evidence supporting bidirectional causal links between hyperthyroidism and GERD or esophageal cancer, reinforcing and extending observations from previous epidemiological studies by demonstrating causality at the genetic level and shedding light on underlying susceptibility mechanisms.

Zhang et al. conducted a two-sample Mendelian randomization study that identified a causal relationship between hypothyroidism and gastric cancer, suggesting that hypothyroidism may be associated with a reduced risk of developing gastric cancer. However, the precise mechanisms underlying this association remain unclear. These findings offer new insights into the etiology and pathogenesis of gastric cancer, though further validation through basic experimental studies is required.

The study performed by Chen et al. provides a broader metabolic context by examining the role of normal-range FT4 variation in the development of biliary tract cancer (BTC). They demonstrated that higher FT4 levels are associated with a lower risk of BTC, and this protective effect is partly mediated by metabolic syndrome (MetS) and waist circumference (WC). This mediation points to an endocrine–metabolic–oncogenic axis, suggesting that even within the euthyroid range, thyroid hormones modulate cancer risk through systemic metabolic regulation. Importantly, these findings align with previous research highlighting the role of thyroid hormones in hepatic lipid metabolism and insulin sensitivity, both of which are established contributors to hepatobiliary carcinogenesis.

Adding a complementary dimension to the genetic and metabolic insights, De Lima et al. investigated the epigenetic regulation of the thyroid-specific transcription factor FOXE1 in differentiated thyroid cancer (DTC). Their study revealed that promoter hypermethylation of FOXE1 CpG islands leads to downregulation of its mRNA and protein expression in papillary thyroid carcinoma (PTC) samples and cell lines. Importantly, reduced FOXE1 expression correlated with aggressive tumor features, including advanced stage, extrathyroidal extension, older age at diagnosis, and the presence of the BRAFV600E mutation. These findings suggest that epigenetic silencing of FOXE1 may contribute to thyroid tumor progression by impairing normal thyroid differentiation pathways. This work highlights an additional layer of thyroid hormone-related gene regulation impacting cancer biology, bridging genetic, epigenetic, and functional mechanisms that collectively shape thyroid tumor behavior.

Together, these studies deepen our understanding of the diverse and sometimes paradoxical roles that thyroid hormones play in cancer biology, spanning anything from protective to promoting effects depending on the context and cancer type. They underscore the importance of integrating endocrine, metabolic, and genetic perspectives to fully elucidate thyroid hormone actions in oncogenesis, ultimately informing future research directions and therapeutic innovations.
